# Challenges and Innovation for Diagnosing and Treatment of Secondary Progressive Multiple Sclerosis

**DOI:** 10.3390/ijms27104558

**Published:** 2026-05-19

**Authors:** Ekdanai Uawithya, Joshua S. Mytych, Ismail Muwenda, Megan Reidy, Meerah Khan, Yang Mao-Draayer

**Affiliations:** Arthritis and Clinical Immunology Program, Oklahoma Medical Research Foundation, 825 NE 13th Street, Oklahoma City, OK 73104, USAjosh-mytych@omrf.org (J.S.M.); ismail-muwenda@omrf.org (I.M.); megan-reidy@omrf.org (M.R.); meerah-khan@omrf.org (M.K.)

**Keywords:** multiple sclerosis, progressive, treatment

## Abstract

The transition from relapsing–remitting multiple sclerosis (RRMS) to secondary-progressive multiple sclerosis (SPMS) represents an ambiguous transition period characterized by diagnostic delays and a shifting therapeutic window. While inflammatory relapses are well-managed, the underlying neurodegeneration often remains undetected until substantial disability has accrued. This review evaluated the shift from traditional metrics, such as the Expanded Disability Status Scale (EDSS), toward more sensitive, multimodal monitoring strategies. We described characteristic MRI findings in SPMS and addressed the impact of comorbidities that frequently confound the diagnosis of disease transition. Furthermore, we evaluated the predictive potential of emerging fluid biomarkers and gut microbial signatures in identifying the early RRMS-to-SPMS transition. Finally, we described the current therapeutic landscape and emerging immunomodulatory interventions. Diagnosing SPMS remains a clinical challenge due to comorbidities and the lack of a singular definitive marker. Moving toward high-sensitivity imaging and molecular biomarkers is essential for the early initiation of treatments and improved patient outcomes.

## 1. Introduction

Multiple sclerosis (MS) is an autoimmune, demyelinating disease of the central nervous system (CNS). The presence and timeline of acute exacerbations or progressive functional decline vary considerably among MS patients. Lublin et al. were the first to standardize diagnostic terminology in 1996. Relapsing–remitting MS (RRMS) is defined as discrete periods of exacerbation or relapse, with no interim disease progression. As a result, patients may fully recover from relapse events or may have residual deficits from these exacerbations. Primary-progressive MS (PPMS) is defined by steady disease progression from onset and may include periods of stable disease or transient symptomatic improvement. In contrast, secondary-progressive MS (SPMS) is characterized by a disease course that begins as RRMS but eventually transitions to disease progression 10–25 years after disease onset [1].

While SPMS has long been considered a distinct clinical phenotype with a unique natural history, pathophysiology, and treatment response compared with other MS phenotypes, there remain a relative lack of clinically applicable guidelines for the prompt diagnosis of SPMS as patients transition from RRMS. In the absence of disease-modifying therapies (DMTs), this transition to SPMS occurs within 10 years in 50% and within 25 years in 90% of RRMS patients [2].

### Narrative Review and Data Synthesis Methods

References for this narrative review were identified through a comprehensive search of the PubMed and Scopus databases conducted between August 2025 and March 2026. The search utilized terms such as ‘Progressive Multiple Sclerosis’, ‘Secondary Progressive Multiple Sclerosis’, ‘Smoldering MS’, ‘PIRA’, ‘Biomarkers’, and ‘Disease-Modifying Therapies’. We reviewed literature published from 1983 through February 2026.

Articles were selected based on their relevance to the diagnostic challenges, imaging advancements, and therapeutic landscape of SPMS. Inclusion criteria focused on peer-reviewed original research, clinical trials, late-breaking data from major international conferences, and recent meta-analyses. Exclusion criteria included studies published in languages other than English and abstracts without full-text availability.

## 2. Clinical Measures of Disability

A variety of clinical disability severity scores have been proposed for the recognition and surveillance of progression in MS. The Expanded Disability Status Scale (EDSS) was developed in the 1950s and has long been the standard for assessing MS progression in clinical trials [3]. The EDSS assesses the following functional systems: pyramidal, brainstem, sensory, bowel and bladder, visual, cerebral, cerebellar, and other. Criticisms of this modality include its emphasis on physical disability, insensitivity to small changes in disease progression as overall disability increases, and its significant interrater variability [4].

In a major 2002 study by Cohen et al., EDSS was inferior to the Multiple Sclerosis Functional Composite (MSFC) in detecting disease progression in patients with SPMS [5]. The MSFC was first outlined in the late 1990s and is considered a more quantitative, psychometrically sound approach to monitoring [6]. It consists of three assessments: a Timed 25-foot Walk (T25-FW), a 9-Hole Peg Test (9HPT), and a paced auditory serial addition test (PASAT). The MSFC assessment of cognition and upper-extremity function provides valuable information not available from the EDSS. Moreover, MSFC scores are strongly correlated with validated measures of quality of life [7]. The major limitation of the MSFC involves the steep learning curve required for patients to perform the various assessments [8].

Additional modalities utilize new composites and technologies to enhance sensitivity. CombiWISE, which combines 58 outcomes from progressive MS trials, is over twice as sensitive as the EDSS when it comes to detecting disease progression [9]. While this modality may prove too unwieldy in clinical practice due to the prohibitive time burden of data collection and the computational complexity required to score the composite in a standard patient encounter, its benefits in clinical trials are promising. Similarly, the Multiple Sclerosis Performance Test (MSPT), developed by Rudick and colleagues at the Cleveland Clinic, addresses the standardization issues inherent in modalities such as the EDSS and MSFC by leveraging modern technological advances, such as neuroperformance module on a tablet [10].

Another key challenge is distinguishing disease progression from normal aging. The Age-Related Multiple Sclerosis Severity (ARMSS) score addresses this by adjusting a patient’s disability score based on age and ranking it against an extensive database of individuals with MS of the same age [11]. However, because it is based on the EDSS, the ARMSS tool also inherits the parent scale’s weaknesses [12].

These metric modalities are also foundational for defining the transition to SPMS. Lorscheider et al., for example, utilized a large global cohort to evaluate and validate objective SPMS definitions [13]. Their resulting criteria employ a three-stratum algorithm for minimum required progression, a 3-month confirmatory period, and a minimum EDSS score of 4. This objective framework provides a practical tool for clinicians to reduce diagnostic uncertainty in patients regarding SPMS-specific treatments or trials.

Building on these definitions, prognostic tools have been developed to predict the risk of conversion to SPMS [14,15]. The Disease duration, Age at disease onset, Age, and EDSS scores (DAAE) score is a more recent clinical instrument for predicting a patient’s five-year risk of conversion [16,17]. Subsequent international validation studies have yielded consistent and promising results, demonstrating that higher DAAE are proportionally associated with an increased risk of conversion to SPMS across diverse patient datasets. Furthermore, to capture the silent progression that often bypasses standard physical examinations, functional assessments such as the Symbol Digit Modalities Test (SDMT) are increasingly used to detect early cognitive decline associated with disease worsening [18].

Finally, while clinician-administered scales provide objective data, they often fail to capture the subjective burden of disease. Patient-Reported Outcome Measures (PROMs) are indispensable for quantifying patients’ perceptions of health status. Validated tools such as the Multiple Sclerosis Impact Scale (MSIS-29) [19] and the Multiple Sclerosis Quality of Life (MSQOL-54) [20] provide crucial insights into the actual impact of SPMS on daily living that clinical metrics alone may miss [21].

Ultimately, no single metric can capture the nature of SPMS progression. While high-density composites such as CombiWISE and MSPT offer superior sensitivity in research settings, they remain logistically challenging for routine care. In clinical practice, a multimodal approach is recommended. The EDSS remains necessary for regulatory and insurance purposes. Still, it should be supplemented by other testing modalities, such as the Timed 25-Foot Walk, the 9-Hole Peg Test, and the SDMT. Integrating these tools enables comprehensive disease monitoring that is rigorous enough to detect progression and compatible with clinical workflows.

## 3. Diagnostic Challenges and Clinical Measures Are Insufficient

The clinical landscape of MS has been shifted recently in how disability accumulation is understood, and moving away from purely relapse theory, which recognizes the early, hard-to-detect onset of neurodegeneration. This shift centers on the distinction between two ways disability can accumulate. Relapse-Associated Worsening (RAW) refers to disability accrual occurring shortly after a clinical relapse. In contrast, Progression Independent of Relapse Activity (PIRA) refers to confirmed worsening in the absence of recent relapse. Both are clinical definitions that do not reflect MRI inflammatory activity, molecular, or pathological changes.

Emerging data suggest that PIRA is not a late-stage phenomenon but a process that begins early in the RRMS phase and evolves throughout the disease course [22]. Kappos et al. identified PIRA as the primary driver of confirmed disability accumulation, accounting for up to 89.1% of events in patients on high-efficacy therapy [23]. However, recent methodological analyses [24] suggest that even these figures may be conservative. Traditional definitions of PIRA, which require broad, relapse-free intervals, tend to be less sensitive when relapse rates are higher, potentially creating a bias that underestimates the true impact of treatment on progression. To address this, researchers advocate for identifying PIRA simply as any confirmed disability that is not a RAW event.

**Figure 1:**
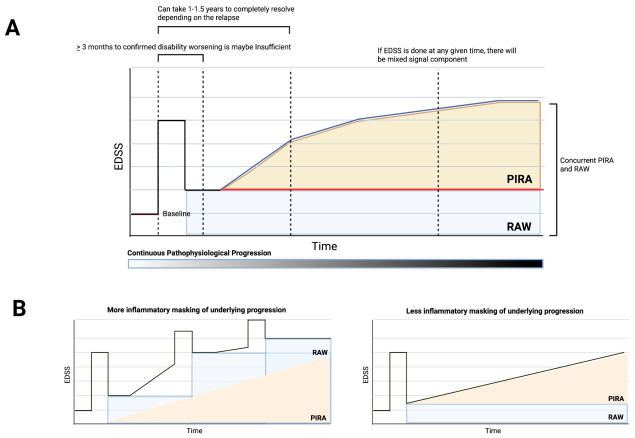
Primary contributors to Disability Accumulation in Multiple Sclerosis. (**A**) Point 1: Disability accumulation in multiple sclerosis is visualized as a composite of non-mutually exclusive drivers. The EDSS at any cross-sectional evaluation point reflects a mixed component of RAW (blue) and PIRA (orange). Point 2: Standard regulatory confirmation periods of ≥3 months are insufficient to distinguish transient inflammatory fluctuations from true progression. We propose that the optimal identification of a pure PIRA signal requires an extended temporal period of 1.0–1.5 years to ensure that the measured disability is independent of transient inflammatory fluctuations and incomplete relapse recovery, particularly in patients with high relapse frequency. Point 3: The gradient bar represents continuous pathophysiological progression. The underlying biological damage is a persistent, background process that precedes clinical detection. (**B**) The visibility of the PIRA signal is dependent on the intensity of the inflammatory process. The use of high-efficacy DMTs leads to a reduction in relapse frequency and severity. By suppressing the relapses, high-efficacy DMTs remove the confounding lingering inflammation from the graph, leading to slow-rising PIRA. Abbreviations: PIRA; Progression Independent of Relapse Activity, RAW; Relapse-Associated Worsening.

Despite advances in detection methods, the clinical distinction between RRMS and SPMS remains unclear. PIRA often occurs concurrently with acute relapses, creating a transition zone where the primary driver of disability shifts from inflammation to neurodegeneration [23]. Furthermore, because PIRA is a retrospective observation requiring months of confirmation, it often serves as a record of past damage rather than a predictive tool (Figure 1). If they are done at any time, there will be a mix of PIRA and RAW components. In reality, the two go hand in hand. Even though EDSS is regularly done at scheduled intervals, such as every 3 months, and rebasing after relapse, there will still be overlap, as one could take up to 1 year to 1.5 years to fully recover from a relapse. Recent studies showed that higher-efficacy therapy has a stronger effect on relapse and less on PIRA (Figure 1).

Complicating this challenge is the absence of a single definitive biomarker of progression. While MRI atrophy and serum neurofilament light chain (sNfL) composites are informative, they currently lack sufficient specificity to serve as a stand-alone diagnostic for SPMS. Consequently, clinicians face a significant dilemma. On one hand, early recognition of progression is critical for initiating neuroprotective interventions and preserving functional reserve. On the other hand, there is substantial hesitancy among both patients and physicians to formally apply the SPMS label. For patients, the diagnosis carries a heavy psychological burden [25]. For physicians, it means limiting therapeutic options due to insurance restrictions and the scarcity of approved treatments for progressive disease [26].

The diagnostic picture is further clouded by pseudoprogression, in which a transient decline due to infection, comorbidities, persistent symptoms such as fatigue, pain, or spasticity can resemble true disease progression. Clinicians must therefore distinguish between reversible confounders, which require symptomatic management, and actual progression, which warrants a change in disease-modifying therapy.

## 4. Comorbidities

Just as a variety of non-MS factors can confound the clinical picture in the setting of suspected relapse, a variety of potentially addressable comorbidities can resemble clinical progression due to MS [27]. Comorbid conditions affecting perceived disease progression in MS patients run the gamut from normal aging and sleep disorders to psychiatric illness and chronic diseases.

Primary sleeping disorders and those secondary to other comorbidities are common among general MS patients [28,29]. Changes in sleep quantity and quality directly affect the cyclic regulation of cytokines, leading to immune system dysregulation [30,31]. Notably, low melatonin levels are also associated with immune system dysregulation and may help to explain the relationship between sleep disorders and relapse in MS patients [32,33]. These authors found that the primary driver of variance in sleep problems among MS patients was depression [34].

Psychiatric diagnoses are more common in patients with MS. When compared to matched controls, MS patients had a higher incidence and prevalence of anxiety, depression, bipolar disorder, and schizophrenia [35]. Depression has been associated with relapse severity [36], a relationship exacerbated by concurrent substance use disorder [37]. This complex interplay highlights how psychiatric comorbidities can significantly influence the clinical course and overall burden of MS.

Obesity and its associated comorbidities are associated with increased adiposity, which also affects disease progression and relapses in MS patients. Adipokines secreted by adipose tissue have inflammatory properties that are thought to contribute to immune dysregulation in MS [38]. Moreover, the sequelae of poor diet and obesity are associated with disease progression and relapse. Vascular comorbidities such as diabetes mellitus, hypertension, hypercholesterolemia, and peripheral vascular disease have all been associated with disability progression in MS patients [39]. Furthermore, improved glycemic control through medical management of diabetes has been shown to strengthen MS relapse rates [40].

Beyond metabolic factors, other environmental and lifestyle factors, such as cigarette smoking, could also be a major culprit for the disease progression and relapse [41]. Smoking can activate the innate immune system, leading to widespread inflammation that, in turn, can damage myelin and axons in the central nervous system [42]. Several studies suggested that smoking can facilitate the transformation to a progressive form of multiple sclerosis [43,44,45]. Drug abuse in MS is multifaceted; while cannabis is frequently used for symptomatic relief of spasticity and pain, its chronic misuse has been linked to cognitive decline and psychiatric instability. Furthermore, the misuse of opioids or alcohol can compromise adherence to disease-modifying therapies and potentially accelerate neurodegeneration [46,47].

Musculoskeletal comorbidities are among the most common in MS, including long-standing spasticity, muscle weakness, and gait. Over time, these symptoms could lead to secondary conditions such as chronic lower back pain, hip and knee osteoarthritis, and degenerative disc disease due to compensatory muscle usage. The patients may show progression on the scale due to mechanical joint pain or a hip injury rather than pathological progression. This creates pseudo-progression, which can lead to misidentification of MS stages.

Finally, aging and MS progression share several overlapping mechanisms, making it challenging to distinguish between natural aging and disease-related decline. The aging process leads to immunosenescence, which reduces immune responsiveness and may alter the course of disease inflammation [48]. Structural white matter degeneration and decreased recovery potential following relapses significantly contribute to this overlap [49,50]. The accumulation of plaque could facilitate the transition from RRMS to the progressive form of MS [51].

**Figure 2:**
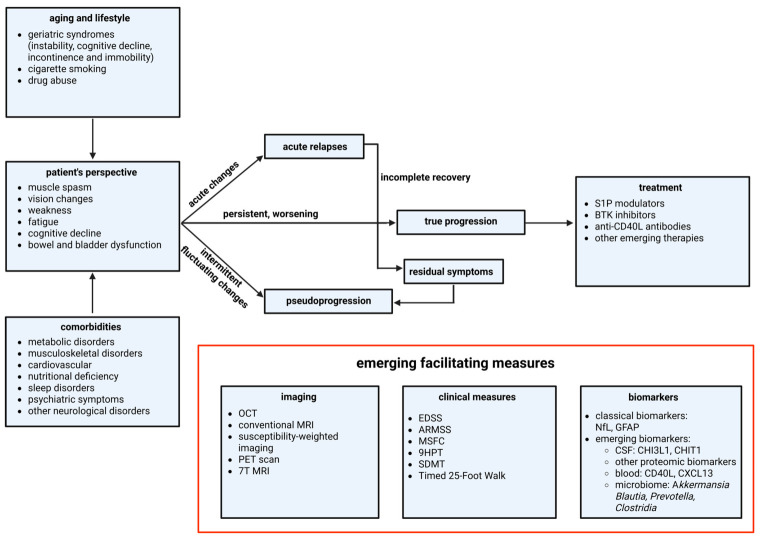
Integrated Diagnostic Framework for Distinguishing True Progression from Pseudoprogression in SPMS. This flowchart outlines the multi-dimensional assessment required to identify true disease progression. The process begins with the Patient’s Perspective on worsening symptoms, which may be confounded by Aging and Lifestyle factors (geriatric syndromes, smoking) or Comorbidities (e.g., cardiovascular or metabolic disorders). Symptom evolution is categorized as acute changes, intermittent fluctuations that can lead to Residual Symptoms, or a steady decline. To differentiate pseudo-progression from true progression, clinicians integrate objective data from Clinical Measures, Biomarkers, and advanced Imaging. Confirmation of true progression guides the selection of disease-modifying Treatments. Abbreviations: 9HPT; 9-Hole Peg Test, ARMSS; Age-Related Multiple Sclerosis Severity, BTK; Bruton’s Tyrosine Kinase, CD40L; Cluster of Differentiation 40 Ligand, CHI3L1; Chitinase-3-like Protein 1, CHIT1; Chitotriosidase-1, CSF; Cerebrospinal Fluid, CXCL13; C-X-C Motif Chemokine Ligand 13, EDSS; Expanded Disability Status Scale, GFAP; Glial Fibrillary Acidic Protein, MRI; Magnetic Resonance Imaging, MSFC; Multiple Sclerosis Functional Composite, NfL; Neurofilament Light Chain, OCT; Optical Coherence Tomography, PET; Positron Emission Tomography, S1P; Sphingosine-1-Phosphate.

The combination of biological aging and MS pathology presents a significant diagnostic challenge, as the clinical presentation of the general aging population frequently mimics the features of progressive MS. Geriatric syndromes such as cognitive decline, gait instability, fatigue, and bladder dysfunction are highly prevalent in the elderly, irrespective of demyelinating disease [52]. For the clinician, distinguishing whether a decline in gait velocity is driven by MS-related spasticity or age-related sarcopenia and osteoarthritis is difficult yet crucial for appropriate management. Similarly, cognitive slowing may be attributed to MS pathology, vascular cognitive impairment, or undiagnosed neurodegenerative comorbidities like Alzheimer’s disease. These presentations overlap, potentially leading to the misattribution of age-related functional decline to MS disease progression [53]. (Figure 2) These comorbidities could make EDSS and other clinical measures appear worse despite the fact that they were not supposed to count in MS-unrelated disability, but practically, EDSS examiners would not be able to tease these out.

## 5. Imaging

Magnetic resonance imaging (MRI) measures are increasingly used in progressive MS trials as proxies for disease progression. A seminal 1999 study by Stevenson et al. demonstrated a correlation between CNS atrophy and EDSS scores in patients with progressive MS. Brain and spinal cord atrophy have largely replaced lesion load as the primary markers in progressive MS trials [54,55]. Ventricular enlargement, brain volume change, and diffusion tensor imaging (DTI) biomarkers have also been studied and, for various reasons, deemed inferior to atrophy as markers of progression [56]. Challenges to standardizing advanced imaging techniques [57] and controlling for other confounders include changes in patient weight, lipid levels, and hydration [58].

Complementing MRI, Optical coherence tomography (OCT) is a newer technique that, over time, can capture changes in the ganglion cell and inner plexiform layer of the retina (RNFL) and macular ganglion cell-inner plexiform layer (GCIPL) thicknesses. Retinal layer atrophy documented via OCT is associated with whole-brain atrophy in MS patients, a relationship that is particularly strong in progressive MS compared to RRMS [59]. The baseline thickness and the annualized rate of retinal layer thinning could also be strong predictors of global disability progression [60,61].

In recent years, the concept of “smoldering MS” has emerged to describe patients who experience progressive disease worsening despite stable inflammatory parameters. Smoldering MS is characterized by compartmentalized inflammation within the CNS, making traditional disease-modifying therapies less effective due to the presence of the blood–brain barrier [62,63].

Recent advances in neuroimaging have been instrumental in refining our understanding of smoldering MS. Using conventional MRI, it is possible to identify slowly expanding lesions (SELs), defined as pre-existing T2-hyperintense white matter lesions that demonstrate a slow, constant expansion over serial imaging studies [64]. Although SEL visualization only requires conventional MRI, SEL requires a long observation period and at least 3 scans to measure the lesion’s activity indirectly [65].

The clinical relevance of SELs is significant. Preziosa et al. established that a higher baseline proportion of SELs is an independent predictor of long-term disability worsening. Notably, more severe microstructural tissue damage within these lesions predicts conversion to SPMS [66]. Complementing this metric, paramagnetic rim lesions (PRLs) are another imaging marker that directly visualizes iron accumulation within activated microglia and macrophages at the lesion edge. PRL could be visualized in multiple imaging modalities. While 7T MRI is considered the standard for visualizing PRLs, Susceptibility-Weighted Imaging (SWI) is a widely used, accessible technique that provides precise, quantitative measures of tissue magnetic susceptibility [67].

Although SEL and PRL are distinct imaging markers, they can colocalize. Elliot et al. demonstrated that 39.5% of chronic PRLs also met the criteria for SEL, whereas 17.2% of SELs had PRL [68]. The paramagnetic rim is also not a permanent feature and tends to diminish as lesions age. This process is thought to reflect a decrease in chronic inflammatory activity at the lesion’s edge, which could be explained by microglia becoming less active and losing their iron content over time [69].

Emerging modalities include positron emission tomography (PET), which visualizes neuroinflammation by targeting the 18 kDa translocator protein (TSPO), a marker expressed in active microglia [70]. This technique shows promise in differentiating SPMS from RRMS, though its high cost limits routine use [71,72].

Finally, in SPMS, spinal cord lesions remain a critical prognostic indicator. These lesions typically reflect chronic degenerative processes, such as demyelination and axonal loss, rather than acute inflammation and often lack gadolinium enhancement on MRI scans [73]. Studies have identified critical spinal cord lesions in SPMS that are highly predictive of disease progression, located in the lateral columns of the spinal cord and appear as prominent, focal areas of atrophy [74]. Beyond the presence of lesions, spinal cord atrophy, specifically in the upper cervical cord, is detectable on MRI scans up to 4 years before the clinical criteria for SPMS are met. Additionally, it serves as a powerful prognostic marker for the progression to SPMS [75].

## 6. Biomarkers of Progression

Numerous patient- and modality-specific factors can affect the specificity of MRI changes in relation to disease progression. Depending on the setting, MRIs can also carry accessibility challenges for MS patients. Thus, recent research has focused on biomarkers of disease activity (see Table 1). While much of the literature focuses on RRMS, there is likely considerable overlap between biomarkers of acute relapses and those of subacute or chronic disease progression observed in SPMS.

Historically, cerebrospinal fluid (CSF) IgG and oligoclonal band levels have been the gold standard for clinically defining MS and although diagnostically crucial, they are poor indicators of relapse [142]. While a significant effort has focused on neurofilament light (NfL) as a potential predictor of disease activity in MS, it is also elevated in other conditions, including Parkinson’s [143,144] and Alzheimer’s [145], and is affected by changes to body mass index [89] and normal aging [146]. Additionally, NfL’s clinical success has been variable, with a comprehensive review of investigational new drug (INDs) submitted to the U.S. Food and Drug Administration over the past 20 years showing that only approximately 50% of studies found NfL to be correlated with investigational drug treatment [147]. Thus, more work is needed to develop accurate biomarkers, particularly those that represent patients’ RRMS-to-SPMS transition.

Traditional biomarkers for MS, such as NfL and glial fibrillary acidic protein (GFAP), and CXCL13 have provided valuable insights into systemic inflammation. However, their utility is limited by a lack of disease specificity and a detection gap that occurs when a restored BBB masks compartmentalized CNS inflammation [148,149]. However, in an effect that is not specific to neurodegeneration, the serum and CSF biomarkers, NfL [150,151] and the chemokine CXCL13 [100,152] are elevated during relapse.

Recent proteomic and longitudinal analyses have shown that while NfL and CXCL13 remain good markers for tracking current disease activity and relapses, they are less effective at predicting long-term outcomes. Instead, the researchers found several promising biomarkers, such as sCD27, sCD40L and chitinase-3-like protein 1 (CHI3L1) levels, correlate strongly with smoldering inflammation and clinical deterioration (Table 1).

Soluble CD27 (sCD27) in CSF has been discovered as a robust biomarker of intrathecal T-cell activation and correlates significantly with NfL levels and axonal damage in progressive MS cohorts. While membrane-bound CD27 is a co-stimulatory receptor for T, B, and NK cells [118,153,154], sCD27 is secreted primarily by T cells during activation [155,156], and serves as a biomarker of intrathecal T cell activation [157], blood levels do not reliably distinguish MS patients [122,158]. sCD27 captures the residual inflammation that persists behind the BBB and reflects long-term disability outcomes [159].

Similarly, increases in serum and CSF GFAP [160] reflect reactive astrogliosis and mechanical injury to the astrocyte cytoskeleton, while decreases in serum response gene to complement 32 (RGC-32), a cell cycle regulator and promoter of Th17 activity, have been associated with response to Glatiramer acetate in RRMS patients [134]. Despite these associations, the diagnostic utility of these markers remains unclear due to confounding variables. Consequently, while these traditional markers have laid the groundwork for monitoring neuroinflammation, there remains a critical need for more refined tools that can accurately distinguish the progression.

Our previous work also led to the discovery of T cell costimulatory molecule sCD40L as a predictive biomarker for MS disease progression [161]. The interaction between CD40, a membrane-bound costimulatory protein expressed on antigen-presenting cells, and CD40L, expressed on activated CD4+ T cells, is a critical co-stimulatory signal required for robust innate and adaptive immune activation [162]. Identification of CD40+ B cells within inflammatory lesions in MS autopsy brain tissue suggests that T cell and B cell interactions mediated by the CD40-CD40L pathway could contribute to MS pathology [163].

The Multiple Sclerosis Disease Activity (MSDA) Test is a commercially available serum-based proteomic assay that quantifies multiple protein markers as currently its utility is limited to relapsing MS. The clinical validation of the MSDA test was performed by correlating its disease activity score with established radiographic and clinical endpoints of inflammatory disease activity; no markers for progressive disease have been validated [164].

Promising advances in the field are focused on alternative markers of disease progression, including microbial signatures, with one pediatric study showing an association between MS and the absence of *Fusobacteria* [165]. Additional studies have shown that, compared with RRMS, SPMS patients are enriched for *Akkermansia* and *Blautia* and depleted of *Prevotella* and *Clostridia*, among others [166,167,168,169]. Further confounding factors include the distinction between progressive and non-progressive phases of MS. Specifically, it is critical to determine whether microbial community shifts function as active drivers of the disease course or are merely secondary bystanders of progression. A recent longitudinal study by Montgomery et al. demonstrated that *Akkermansia muciniphila* and *Prevotella* species are elevated in progressive MS patients and are correlated with changes to a key microbial metabolite, vitamin K [166]. This study reflected a 5-year longitudinal follow-up of progressive MS patients and identified a decreased prevalence of species that produce anti-inflammatory vitamin K and short-chain fatty acids, including *A. muciniphila, Lachnospiracea,* and *Oscillospiraceae*. The study suggests that a selective decrease in these species might promote the acute, damaging inflammation during progressive disease. Later on a 2-year longitudinal follow-up study of patients with progressive MS, Schwerdtfeger et al. found a similar decrease in short-chain fatty acid-producing species, including *Akkermansia* and *Lachnospiraceae* [167]. Efforts to attribute microbial changes to EDSS and patient outcomes have been targeted at single-timepoint or cross-sectional study designs, with one recent meta-analysis highlighting that of 23 studies that met inclusion criteria from 2011 to 2024, 4 were longitudinal, with the remaining 19 being cross-sectional [168]. The connection between gastrointestinal colonization and neurodegeneration is an active area of research within the field of multiple sclerosis, and although not yet standardized into clinical practice, it has already shown potential to improve patient outcomes [170,171].

## 7. Disease-Modifying Treatments

### 7.1. Established Therapies

Two relatively recent advances in disease-modifying therapy (DMT) for MS have been in the anti-CD20 and sphingosine-1-phosphate (S1P) modulator classes. Anti-CD20 medications include the first-generation rituximab which, while widely utilized, remains off-label in the US for the treatment of MS [172], and the second-generation humanized anti-CD20 ocrelizumab, was approved as the first medication for PPMS [173]. These medications primarily target B cells, and ocrelizumab was designed to enhance cell death in CD20-expressing cells. It was approved for PPMS following a trial that demonstrated reduced confirmed disability progression at 3 and 6 months, slower changes in timed walks, and reduced brain volume loss [173]. Ocrelizumab initiation starts with two 300 mg intravenous infusions given 2 weeks apart, followed by 600 mg doses every 6 months.

For SPMS, the S1P modulators fingolimod and siponimod have proven promising. Fingolimod works on S1P receptors 1, 3, 4, and 5, whereas siponimod targets receptors 1 and 5 [174]. Fingolimod-specific data primarily come from the 2008—2011 INFORMS trial, which showed limited benefit of fingolimod for progressive disease [175]. The EXPAND trial, conducted from 2013 to 2015, demonstrated that siponimod reduced the risk of disability progression and brain volume loss in patients with SPMS. Adverse effects of siponimod in the EXPAND trial included lymphopenia and infection, elevated liver transaminase values, bradycardia, and bradyarrhythmia [176].

### 7.2. Late-Stage Investigational Approaches

Bruton’s tyrosine kinase (BTK) is a critical protein in signaling pathways of immune cells, such as B cells and microglia, implicated in MS pathology. In progressive MS, BTK-expressing immune populations have been observed in and around chronic active lesions, consistent with the concept of “smoldering” compartmentalized inflammation that contributes to ongoing demyelination, axonal injury, and neurodegeneration [177]. Activated B cells release pro-inflammatory cytokines and form damaging follicle-like structures in the central nervous system, while microglia activation also drives disease progression. Therapeutically, BTK inhibitors (BTKi) are attractive because they may modulate both peripheral B-cell activation and CNS-compartmentalized innate immunity, thereby potentially impacting both relapses and progression [178].

BTKi is a promising but experimental treatment. Recent Phase III studies showed both the promise of BTK inhibition and the importance of defining progressive MS populations most likely to benefit. However, trial outcomes have diverged across studies. In non-relapsing SPMS, the HERCULES trial evaluated tolebrutinib versus placebo and reported a statistically significant reduction in 6-month confirmed disability, although effects on brain atrophy were not observed [179]. However, safety concerns regarding drug-induced liver injury were notable, with elevated enzymes occurring in 4% of treated patients. In PPMS, however, trial outcomes have diverged across studies. The PERSEUS study, which also evaluated tolebrutinib, did not meet its primary endpoint, prompting the manufacturer to pause PPMS registration efforts [180]. In contrast, the FENtrepid study met its primary endpoint in PPMS, demonstrating fenebrutinib was non-inferior to ocrelizumab in delaying the onset of 12-week composite confirmed disability progression, with a numerical benefit observed as early as week 24 [181].

These different outcomes likely reflect a combination of patient selection, trial design, and drug-specific properties. The success in SPMS, compared with the mixed results in PPMS, suggests that BTK inhibition may be most effective in subpopulations with specific inflammatory profiles rather than in broad progressive cohorts. Furthermore, differences in trial endpoints (12-week vs. 6-month confirmation) and comparators (placebo vs. ocrelizumab) complicate direct comparisons. Finally, while BTK inhibition remains a compelling therapeutic avenue, future success will depend on refining patient selection through biomarkers and optimizing agents for selectivity, CNS penetrance, and safety profiles.

The CD40-CD40L pathway is a potent therapeutic target because it bridges adaptive and innate immunity; CD40L expressed on pathogenic T cells drives B cell and antigen-presenting cell activation, contributing to both peripheral inflammation and CNS compartmentalized disease [163,182]. We conducted an investigator-initiated phase I study using the first-generation anti-CD40L antibody, toralizumab (IDEC-131), and demonstrated a favorable safety profile. We showed by flow cytometry that there was no depletion of lymphocyte subsets. We further demonstrated increases in the CD25+/CD3+ and CD25+/CD4+ ratios, as well as the IL10/IL17 and IL10/MCP1 ratios, indicating a shift toward an anti-inflammatory cytokine response with toralizumab treatment [183].

The focus has since shifted to frexalimab, a second-generation antibody engineered to block CD40L without inducing platelet aggregation or thromboembolism is currently under investigation. In a Phase 2 trial for relapsing MS, frexalimab met its primary endpoint at Week 12, reducing the number of new gadolinium-enhancing T1 lesions by 89% in the high-dose group compared to placebo (adjusted mean lesions: 0.2 vs. 1.4). Long-term data from the 48-week open-label extension demonstrated that 96% of participants in the high-dose arm remained free of new T1 lesions. The annualized relapse rate was exceedingly low at 0.04. Crucially, the drug was well-tolerated with no thromboembolic events reported [130].

Based on these promising results, frexalimab is now being evaluated in a significant global Phase 3 trial, FREVIVA (NCT06141486) [184], for non-relapsing Secondary Progressive Multiple Sclerosis. This study is designed to determine if frexalimab can effectively delay disability progression compared to a placebo. As the trial is ongoing, efficacy and safety data for frexalimab in the SPMS population are not yet available.

### 7.3. Early Experimental Strategies

CAR-T cell therapies are emerging therapies that enable T-cell activation and enable targeting of cells with downregulated MHC class I expression. CAR-T cell therapies were primarily used in multiple hematologic malignancies; however, some subtypes showed benefits in MS. Specifically, CAR-Tregs and anti-CD19 CAR-T cells show decreased inflammation and promotion of remyelination in the experimental autoimmune encephalomyelitis model [185]. CAR-T cells have also been tested in several neuroimmunological diseases, including neuromyelitis optica spectrum disorder [186], and myelin oligodendrocyte glycoprotein antibody-associated disease (MOGAD) [187].

For MS patients, CAR-T cell therapy is still in its early phases of development. However, the first publicly available study on MS showed promising results [188]. The study recruited two patients with progressive MS treated with KYV-101, a CD19 CAR-T cell therapy. One patient developed cytokine release syndrome and a transient increase in transaminase levels. Her EDSS and MRIs were stable at 8 weeks. The second patient developed a cutaneous lesion post-infusion. Her EDSS and MRI remained stable throughout the course at 48 weeks of follow-up. Further preliminary research into different CAR-T cell therapies has emerged from a Phase 1 study of Eque-cel (CT103A), which targets the B-cell maturation antigen (BCMA) on plasma cells. In a cohort of 5 patients (4 SPMS and 1 PPMS) followed for an average of 5.1 months, the average EDSS score significantly improved from 6.2 to 5.0, with improvements across all testing modalities and minimal adverse effects [189].

Although CAR-T cell therapies have shown significant benefits in initial studies, their associated toxicities cannot be overlooked. The two major ones are cytokine release syndrome (CRS), which can be presented with symptoms ranging from mild fever to severe shock, and immune effector cell-associated neurotoxicity syndrome (ICANS), which manifests as a spectrum of neurologic symptoms, including confusion, aphasia, delirium, seizures, and, in severe cases, cerebral edema [190,191].

Beyond these successes, the field has also witnessed setbacks, most notably with clemastine fumarate. Originally developed as an over-the-counter antihistamine with muscarinic receptor-blocking properties, it attracted attention as a potential therapy for multiple sclerosis because of its ability to promote oligodendrocyte differentiation and remyelination in preclinical studies [192,193,194] and in the ReBUILD Phase II trial (NCT02040298) [195]. In the phase II ReBUILD trial, it reduced visual evoked potential (VEP) P100 latencies by 1.7 ms per eye (*p* = 0.0048) in patients with relapsing–remitting MS with chronic demyelinating optic neuropathy, suggesting the occurrence of functional recovery through remyelination [195]. These findings provided the rationale for testing clemastine in the TRAP-MS platform trial, which was designed to test a biomarker-guided combination of therapies in patients with progressive MS who were accumulating disability independently of relapses [196].

Contrary to expectations, the clemastine arm of TRAP-MS was terminated early after a third of participants met the prespecified safety-stopping criteria, and most treated patients showed significantly more rapid worsening than that at baseline or compared with patients in other TRAP-MS treatment arms. Follow-up analyses revealed that clemastine potentiates purinergic P2RX7 signaling, enhancing inflammasome activation and inducing pyroptotic cell death in macrophages and oligodendrocytes, thereby worsening CNS injury in a pro-inflammatory environment [196].

## 8. Conclusions

The ambiguity that comes with diagnosing SPMS results in a substantial delay in definitively characterizing patients as having progressive disease, often after they have accumulated substantial disability [197]. Historically, clinicians may have exhibited appropriate hesitancy to label patients as having a condition for which there are limited treatment options, but as new therapies are being developed that may offer neuroprotective benefits for SPMS patients, early identification of those patients who may benefit from existing treatments or participate in clinical trials for new agents becomes increasingly important. This review explored the comorbidities associated with SPMS, metrics for monitoring disability progression, relevant imaging modalities, and promising fluid biomarkers, before then providing a detailed analysis of the current treatment landscape.

Our current understanding of the pathophysiology of multiple sclerosis remains incomplete. Yet the paradigm is shifting from a dichotomous view of relapsing versus progressive disease toward a continuum of immune-mediated injury. We have outlined advances in the field that inform our understanding of comorbidities, the design of high-sensitivity disability metrics, progression-specific biomarkers, advanced imaging, and new DMTs. However, significant hurdles remain. The field must address the diagnostic dilemma posed by the early superimposition of progression on relapses and the current lack of a definitive marker for the transition to SPMS. Future research should prioritize the development of biomarkers that can distinguish actual biological progression from pseudoprogression, therefore enabling clinicians to overcome diagnostic uncertainty. We hope that bridging these knowledge and clinical management gaps over the next decade will facilitate earlier, more targeted interventions for patients entering the progressive phase of the disease.

## Figures and Tables

**Table 1:** Overview of potential biomarkers in MS. This table summarizes key biomarkers, including their biological role, level of evidence, and advantages or disadvantages in MS. These findings provide a framework for comparing traditional, well-established biomarkers to those more recently identified.

Biomarker	Biological Role	Localization (Measuring Blood Relative to CSF)	Major Cell Type Responsible for Production	Level of Evidence	Clinical Utility	Major Limitations	Major Advantages
oligoclonal Bands (antibody-rich protein bands in the CSF)	part of gold standard for MS diagnosis [76]	CSF	plasma cells [77]	strong (diagnostic) weak (pathogenic mechanism)	MS diagnostic criteria	persist despite B-cell depletion, does not reliably predict patient progression or disease course, may bind host antigens to promote debris clearance [78,79,80,81]	gold standard for diagnosis, highly specific to MS [82]
sNfL	cytoskeletal protein in axons [83]	CSF > blood [84]	neurological (neuron/axon) [83]	strong [85]	correlates to EDSS, relapse, and MRI lesions [86,87]	confounding factors (age, BMI, injury) [88,89]; require high-sensitivity assays [90]; levels fluctuate, need to re-measure for baseline [91,92]	when age- and weight-controlled, can be a good marker of inflammation [93]; may assist in identifying PIRA [85]
CXCL13	recruitment of B and T cells to the CNS [94]	CSF > blood [95]	immune/stromal cells [96]	strong [97,98,99]	correlates to EDSS, relapse, and MRI lesions [100,101]	no clear, standardized diagnostic threshold	may assist in identifying PIRA [102]
Glial Fibrillary Acidic Protein (GFAP)	intermediate filament protein [103]	CSF > blood [104]	astrocytes [105]	strong [106]	correlates to EDSS, disease progression, drug response [104,107,108]	no clear, standardized diagnostic threshold	may assist in identifying PIRA [102,109]
CHI3L1 (Chitinase-3-like-1)	unclear biological role. Varied functions—apoptosis, inflammation [110]	CSF, blood [111]	mainly astrocytes in CNS, peripherally other cells can [112,113,114]	growing	correlates to EDSS, cognitive measures, and MRI [111,115]	no clear, standardized diagnostic threshold; relatively new marker, may have cofounders fluctuates with age [116]	none
sCD27	co-stimulatory molecule, denotes memory subsets [117,118,119]	blood > CSF [120]	activated T cells [117]	growing	unclear [121], correlates to relapse [122]	no clear, standardized diagnostic threshold	none
sCD40L	co-stimulatory molecule [123]	unclear, detectable in both [124,125,126]	activated T cells and platelets [127]	growing	correlates to drug response and disease progression [128]	no clear, standardized diagnostic threshold; first-generation antibodies caused thromboembolic events [129]	targetable—passed phase II clinical trials [130]
RGC-32 (response gene to complement 32)	positive regulator of cell cycle [131]	unclear, blood > CSF due to lack of CSF correlation data [132]	astrocytes [133]	growing	correlates to EDSS, drug response, and co-localizes in plaques [132,134]	no clear, standardized diagnostic threshold; relatively new marker; dual role—both pro- and anti-inflammatory [133]	none
CHIT1 (Chitotriosidase-1)	hydrolyze chitin [135]	unclear, CSF may be preferred [136]	neutrophils, macrophages, and microglia [137,138]	growing	correlates to EDSS, microglial activation markers, and demyelinating lesions and progression [136,139]	no clear, standardized diagnostic threshold; relatively new marker with limited clinical studies, may have cofounders—fluctuates with age [139,140,141]	may track with microglial activation and disease progression [139]

## Data Availability

No new data were created or analyzed in this study.
